# Immunizing the Immune: Can We Overcome Influenza’s Most Formidable Challenge?

**DOI:** 10.3390/vaccines6040068

**Published:** 2018-09-22

**Authors:** Ali H. Ellebedy

**Affiliations:** Division of Immunobiology, Department of Pathology and Immunology, Washington University School of Medicine, 660 S. Euclid Avenue, St. Louis, MO 63110, USA; ellebedy@wustl.edu

**Keywords:** pre-existing immunity, influenza, B cell responses, protection threshold

## Abstract

The first human influenza virus was isolated more than 85 years ago, and several vaccine candidates were developed and tested soon after. Yet, controlling infections mediated by this respiratory pathogen continues to present a formidable challenge. Development of an effective influenza vaccine has been undermined by the dynamic nature of influenza viruses: these viruses have the unique capacity to escape pre-existing immunity. In this perspective, I highlight pre-existing immunity as a different, but related, hurdle that may actually lessen the effectiveness of influenza vaccine-induced immune responses. Specifically, I discuss the impact of pre-existing immunity on the generation of de novo B cell responses to influenza vaccination. As the influenza virus changes its major antigenic determinants, it creates new ones in the process. Our immune system adapts by targeting the new determinants. However, pre-existing antibodies and memory B cells interfere with the generation of de novo responses against these newly formed epitopes, rendering vaccines less effective. Overcoming such interference is essential for the development of more effective influenza vaccines.

## 1. Introduction

There is no better way to describe influenza viruses than the endlessly vivid description by Thomas Francis in his article “On the Doctrine of Original Antigenic Sin” [1]:

“INFLUENZA has always been a mixture of romance and terror; of fact and fable; of new and old ideas. Its various popular names: the jolly rant, the new delight, the newe acquayantance, gallants’ disease, the fashionable illness, influenza di freddo or influenza di coeli, la grippe, flu, this virus thing—all indicate a light-hearted annoyance. But interspersed are the tales of damaging experiences.”

The first human influenza virus was isolated more than eight decades ago and the first protective vaccines against influenza were tested in 1940s. Yet, influenza viruses continue to cause up to 500,000 deaths annually [2]. The challenge of developing an effective vaccine for these elusive viruses has been characterized as trying to “catch a moving target”. This is despite the fact that an infection with any influenza strain induces a life-long immunity against that particular strain [3], meaning our immune system is actually equipped to efficiently handle the “target”. However, in the case of influenza viruses, the target itself changes. The process by which influenza viruses gradually metamorphose their proteins to escape neutralizing antibodies is known as antigenic drift [4].

The protective immunity induced by prior influenza exposure is mediated by a shield of neutralizing antibodies directed against the main surface glycoprotein of the virus, the hemagglutinin (HA). A second layer of protection comes from CD8+ T cells that would clear virus infected cells. However, influenza viruses are known to evolve rapidly, with HA being the most variable of the virus proteins. This makes influenza more difficult to target compared to other pathogens for which successful human vaccines have been developed. Influenza vaccines are offered to the public on an annual basis [5]. This is different from vaccination strategies against most other pathogens where the vaccines are given to immunologically naïve populations. The practice of annual influenza immunization was established to make sure that influenza virus strains included in the vaccine match circulating strains. However, given that at some seasons no or minor drift in circulating strains occurs, the strains selected in consecutive seasons are often antigenically related and, in some instances, identical. For example, the strain that represented the H1N1 component of the 2010/11 seasonal vaccine was A/California/7/2009 (H1N1)-like virus, and that strain did not change in influenza vaccine formulations that were offered in the following 6 seasons.

In addition to HA, protective antibody responses that are directed against the other major surface glycoprotein of the virus—neuraminidase or NA—have been reported [6]. Antibody responses to NA are far less characterized compared to those directed against the HA. We know very little about the major antigenic sites on NA and the potential of these sites to drift. In this perspective, I am focusing on the B cell/antibody responses directed against HA. It is also important to mention that I am not discussing here the important roles of CD4+ [7] and CD8+ [8] T cell responses in modulating the immune response elicited by influenza vaccines, as this is outside the scope of this perspective.

## 2. B Cell Response to Influenza Virus Vaccination in Humans

**Figure 1:**
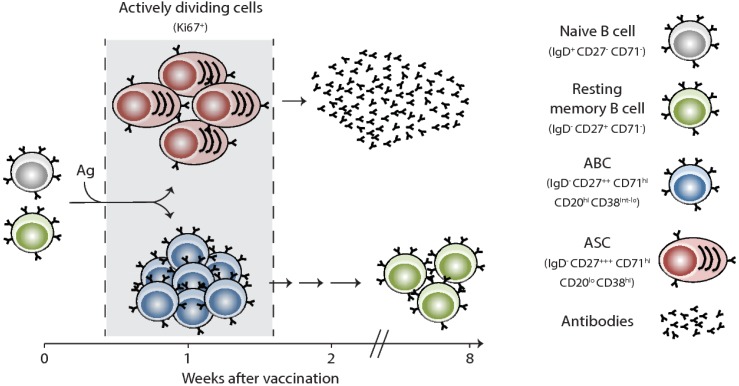
Human B cell responses to seasonal influenza vaccination in humans. A graphical model illustrating the phenotype and kinetics of B cell responses to inactivated seasonal influenza vaccines as detected in human peripheral blood. Ki67 is a nuclear proliferation marker; ABC is activated B cells; ASC is antibody secreting cells or plasmablasts.

Vaccination remains the most successful intervention to prevent influenza virus infection and transmission. Effective vaccines against influenza viruses work by eliciting antibody responses that primarily target the HA. In general, upon initial interactions between vaccine antigens and their corresponding B and cognate CD4+ T cells in the draining lymph nodes, antigen-specific B cells proliferate and undertake one of two fates: (1) rapidly differentiate into extrafollicular short-lived plasmablasts, which provide the first wave of protective antibodies, or (2) migrate to the B-cell follicle and seed a germinal center (GC) reaction [9,10]. In the GC, B cells undergo multiple rounds of proliferation and affinity-based selection. Germinal Center B cells exit the GC reaction either as memory B cells (MBCs) or long-lived plasma cells (LLPCs). In the case of seasonal influenza vaccinations, our knowledge is currently restricted to information obtained via the easily accessible peripheral blood compartment. In blood, influenza vaccine-induced plasmablasts (also termed antibody secreting cells or ASCs) emerge early and can be observed transiently in blood (day 6 or 7 after vaccination) (Figure 1) [11,12,13,14]. These ASCs likely originate from pre-existing MBCs based on their rapid emergence in blood and the fact that they express highly mutated, isotype-switched B cell receptors (BCRs). After seasonal influenza vaccination, there is a correlation between the degree of the plasmablast response and the increase in serum hemagglutination inhibiting (HAI) antibody titers.

Influenza vaccination also induces a B cell subset, termed activated B cells or ABCs, that is phenotypically and transcriptionally distinct from ASCs (Figure 1) [15]. Instead of differentiating into plasmablasts, ABCs are recruited to the memory B cell pool, suggesting that ABCs contain memory B cell precursors. Blood ASC and the ABC populations represent a precious window for us to examine vaccine-induced B cell responses in humans. It remains unclear, however, if any of these responses are GC-derived or if they are entirely generated via the extrafollicular route. It is also unclear how efficiently current seasonal influenza vaccines induce a GC reaction. Another open question is whether any of the vaccine-generated plasmablasts actually end up in the LLPC compartment in the bone marrow. The number and identity of the epitopes (within HA, for example) that are targeted by influenza vaccination-induced plasmablast response are determined by both the precursor frequency of B cells and serum antibody levels that are specific to such epitopes.

## 3. Influenza “Hyper-Seropositive” State and the “Ceiling” Effect

Most adult humans have been exposed—at least once—to influenza virus antigens and.the serological profile for each individual is reflective of their influenza exposure history. This exposure could be in the form of active infection (clinical or subclinical) or in the form of annual or pre-pandemic vaccination. Frequent exposures to multiple complex influenza proteins, such as HA, that share several antigenic epitopes may lead to a build-up of humoral immune memory. This memory is manifested in higher levels of serum antibody titers, which reflect an accumulation of LLPCs and memory B cells directed against these epitopes. Indeed, levels of circulating IgG+ memory B cells directed against antigens included in the seasonal influenza vaccine are 50×, 10× and 5× higher than those directed against the mumps, measles, and rubella antigens, respectively (manuscript in preparation). In areas where annual vaccination against influenza is common, some adults become “hyper-seropositive”. After each additional vaccination, pre-existing antibodies directed against one or more epitopes within the immunization antigen could interfere with the generation of new responses to those epitopes. This interference could potentially take place at multiple stages during the B cell stimulation process. Pre-existing antibodies could play a role in the rapid clearance of the already limited amount of influenza antigens included in the seasonal vaccine. These antibodies could also sterically hinder B cell receptor access to its specific epitope [16,17]. Pre-existing or rapidly secreted antibodies could also prematurely shorten the GC reaction by blocking access to free antigens [18]. An example of interference by pre-existing antibodies with de novo immune responses is what happens in infants. On the one hand, acquisition of maternal antibodies both in utero and via breast milk helps protect neonates against a variety of pathogens, including the influenza virus [19,20]. On the other hand, maternal antibodies are associated with significantly impaired vaccine-induced Ab responses [21]. Thus, the timing of vaccine administration is particularly critical for infants with rapid maternal Ab clearance as delays render them more vulnerable to infection.

**Figure 2:**
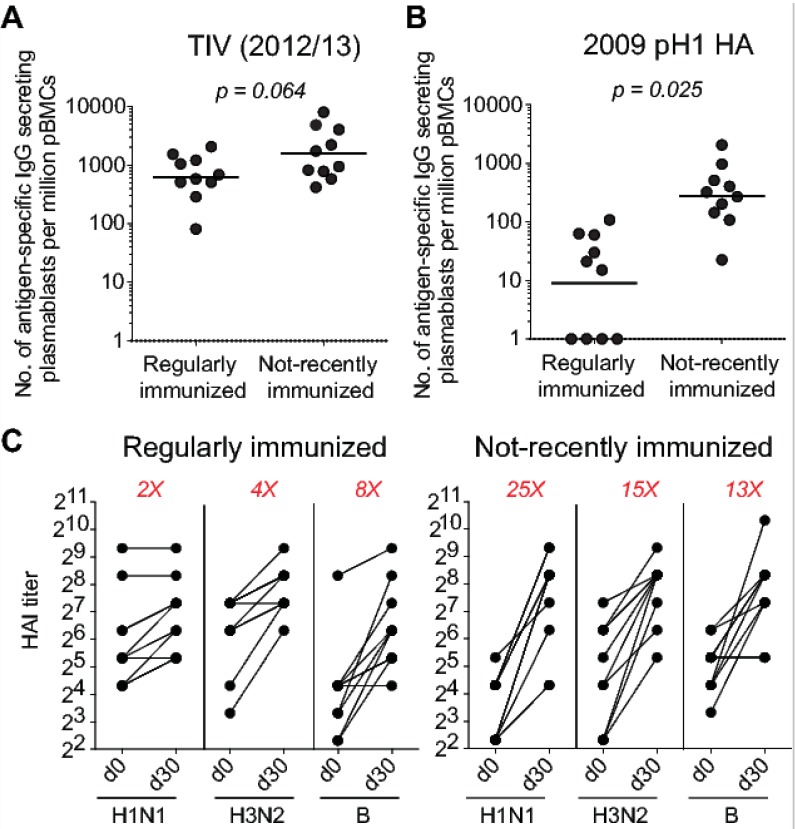
Pre-existing immunity blunts B cell response to seasonal influenza vaccination. Healthy adult volunteers were vaccinated with the 2012/13 inactivated seasonal influenza vaccine (*n* = 20). (**A**) Frequency of IgG-secreting plasmablasts directed against the vaccine antigen in regularly immunized (*n* = 10) and not-recently immunized (*n* = 10) subjects. (**B**) Frequency of IgG-secreting plasmablasts directed against the 2009 pandemic H1 HA (the H1N1 component of the vaccine). (**C**) Serum hemagglutination inhibition titers against the three viral strains contained in the vaccine, A/California/07/2009 (H1N1), A/Victoria/361/2011 (H3N2), and B/Brisbane/60/2008. Titers were measured before and 4 weeks after vaccination. Unpaired Student t tests were used to derive *p* values.

To test the possibility that pre-existing antibodies impact the immune response to influenza vaccination, we recruited and immunized two cohorts of healthy young adults during the 2012/13 influenza season (18–45 years old). The first cohort reported regularly receiving the annual influenza vaccine (*n* = 10), the second cohort reported not having received the influenza vaccination for at least three seasons prior (*n* = 10). Both groups received the inactivated seasonal influenza vaccine. The total vaccine-specific, IgG-secreting, plasmablast response (detected at day 7) in the not-recently vaccinated group was about 3-fold higher than in the regularly vaccinated group (Figure 2A). The plasmablast response against the influenza A H1N1 HA component of the vaccine (A/California/07/2009) was about 25-fold higher in the not-recently immunized group (Figure 2B). These results were consistent with the observed increase in HAI serum antibody titers against the three vaccine strains (Figure 2C). These data are also consistent with multiple reports suggesting a “ceiling” effect that potentially contributes to muted B cell responses following seasonal influenza vaccination in humans [22,23,24,25,26]. The “ceiling” here denotes the level of pre-existing serum antibodies that would block any further boosting following antigen re-exposure.

## 4. “Low-Protective” vs. “High-Protective” Capacity Epitopes

**Figure 3:**
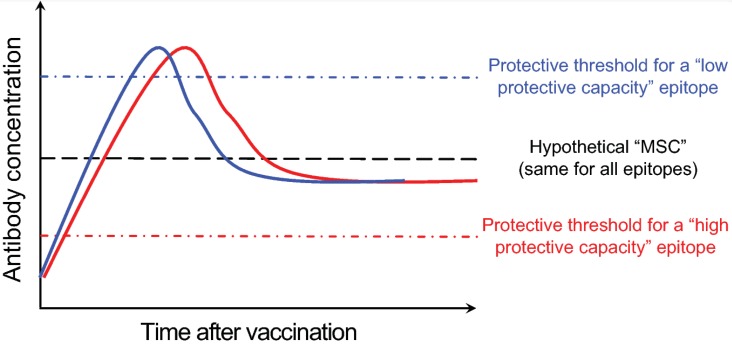
Proposed model for high- and low-protective capacity B cell epitopes. This is a hypothetical plot of serum anti-influenza antibody concentration vs. time after influenza vaccination. Shown are examples of the kinetics of antibody responses directed against two distinct epitopes (blue and red solid lines). Responses against the “red” epitope are protective due to its relatively low protective threshold and vice versa for responses directed against the “blue” epitope. The black dotted line in the middle represents a hypothetical “maximal sustainable concentration” of serum antibodies against any epitope.

More than three decades have passed since the major epitopes targeted by neutralizing antibodies within the influenza HA molecule were defined [3,27,28]. By screening influenza virus escape mutants in the presence of neutralizing murine anti-HA monoclonal antibodies (mAbs), five distinct influenza H1 subtype HA epitopes were identified: Sa, Sb, Ca1, Ca2 and Cb [28]. These epitopes were then mapped to areas around the receptor binding domain within the HA globular head region. Recent work in mice has elegantly analyzed the immunodominance and kinetics of B cell responses directed against these epitopes [29]. In a complex protein like HA, in addition to the more recently characterized epitopes, there are likely other non-neutralizing epitopes [30,31]. However, the degree of in vivo protective capacity afforded by antibodies targeting these epitopes remains unclear. It is likely that the in vivo protective capacity will not be equivalent among antibodies directed against all epitopes, rather it will depend on factors such as the exact location of the epitope relative to the sialic acid receptor binding domain and the relative accessibility of the epitope on the virion surface. This is supported by the observation that monoclonal antibodies that block hemagglutination (HAI+) and neutralize influenza viruses in vitro (microneutralization or MN+) can more efficiently protect mice against H7N9 virus challenge in comparison to HAI- MN+ and HAI- MN- anti-H7 HA monoclonal antibodies [32]. One could then broadly categorize influenza B cell epitopes into “low-protective” and “high-protective” capacity ones, defined by the amount of antibodies directed against the epitope necessary to attain protection (Figure 3). These categories could be further defined using a “protective threshold”, which would be high for “low-protective” capacity epitopes and vice versa. There is probably a limit to the concentration of antibodies directed against any specific epitope that can be physiologically maintained. I am going to refer to that limit as the maximal sustainable concentration or MSC. Given that the mechanisms and cells (LLPCs) responsible for maintaining long-term serum antibody titers against different epitopes are the same, it would be fair to assume that the MSC is similar for all epitopes. Using the simplified model shown in Figure 3 enables us then to infer the long-term protective capacity of antibodies directed against a particular epitope by determining the “protective threshold” of that epitope relative to the MSC.

Given the hypothetical nature of the proposed model, it will be essential to experimentally test its validity by deriving actual values for specific epitopes. Such an approach will reveal which epitopes we should refocus the immune response towards. It is also critical to note that “hyper-seropositive” individuals are not necessarily immune to all influenza virus infections. Rather, immunity depends on the degree to which one’s serological profile matches the infecting virus strain. For example, when an individual possesses high pre-existing antibody titers against a “low-protective” capacity epitope on the influenza virus vaccine strain, these antibodies could hinder the generation sufficient de novo antibody response against a “high-protective” capacity epitope, making this individual more vulnerable to influenza infection.

## 5. The HA Stem as a Universal Vaccine Target: Potential Issues?

**Figure 4:**
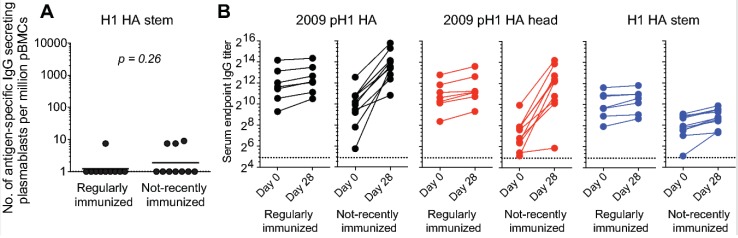
Poor boosting of anti-HA stem antibodies following seasonal vaccination. Healthy adult volunteers were vaccinated with the 2012/13 inactivated seasonal influenza vaccine (*n* = 20). (**A**) Frequency of IgG-secreting plasmablasts directed against the vaccine antigen in regularly immunized (*n* = 10) and not-recently immunized (*n* = 10) subjects. (**B**) Pre- and 28 d post-vaccination serum IgG antibody titers against the 2009 pandemic H1 HA (left, black), pandemic H1 HA head region (middle, red), and H1 stem region (right, blue). Each symbol represents one individual (*n* = 10/group). Unpaired Student t tests were used to derive *p* values.

Compared to HA head epitopes, epitopes in the HA stem region are more highly conserved across different influenza HA subtypes [33,34,35,36], making them attractive influenza vaccine targets. Antibodies targeting the HA stem region can be broadly neutralizing, and thus provide broad anti-influenza immunity. Several distinct epitopes are targeted by broadly neutralizing antibodies within the HA stem region. These epitopes differ in several aspects, including the extent to which they are conserved. In addition, these epitopes may differ in the degree to which their corresponding mAbs efficiently block viral replication in vitro and in vivo, impacting epitope accessibility on the virion surface. Here, I will categorically refer to these as HA stem epitopes, and propose that they are an example of “low-protective” capacity epitopes based on the following observations: (1) in vitro, neutralizing mAbs directed against certain head epitopes more potently block viral replication than their counterparts directed against stem epitopes (unpublished observations); (2) serum antibodies directed against HA stem epitopes are widely prevalent in humans, despite the general population’s overall susceptibility to influenza virus infection [37,38,39]. Pre-existing anti-HA stem serum antibodies could contribute to the blocking of further vaccine-mediated boosting of responses directed against this region. Indeed, in the study shown in Figure 2, we did not observe a significant plasmablast response directed at the stem region of the H1 HA component of the vaccine, even in the cohort that had not recently received an influenza vaccination and responded robustly to all three components of the vaccine (Figure 4A). The increase in serum IgG antibody titers against H1 HA responses were mainly directed against the head region of the HA (Figure 4B). These observations lead us to the question of whether pre-existing antibodies block significant increases in anti-HA stem antibody titers. If this is indeed the case, then the next step is to determine whether there are potential vaccine candidates that can overcome this hurdle.

## 6. Overcoming the Hurdle of Pre-Existing Immunity to Influenza

### 6.1. Increasing the Vaccination Dose

Perhaps the simplest way to overcome interference by pre-existing antibodies is to increase the dose of the vaccine. This strategy has already been successfully deployed with seasonal influenza vaccines tailored for the elderly [40,41]. The high-dose vaccine formulation contains four times the amount of antigen contained in regular seasonal influenza vaccine. The high-dose vaccine is 24.2% more effective in preventing infection in adults aged 65 and older relative to a standard-dose vaccine. It is also associated with a lower risk of hospital admissions compared with the standard dose [40,41].

### 6.2. Adjuvants

The poor immunogenicity of split and subunit influenza vaccines (especially with antigens derived from avian influenza viruses) necessitated the use of adjuvants in combination with these vaccines [42,43,44]. Currently, oil-in-water emulsion adjuvants, such as MF59 and AS03, are the main class of adjuvants being used with human influenza vaccines [44,45,46]. These adjuvants were empirically developed and the mechanisms through which they potentiate immune responses are not fully understood. Immunization of healthy subjects with inactivated pre-pandemic H5N1 vaccine in combination with AS03 leads to recruitment and stimulation of both cross-reactive memory B cells and naïve B cells (manuscript in preparation). The cross-reactive responses were entirely directed against epitopes within the HA stem region, which is consistent with previously published reports [34,35]. The naïve-derived responses, on the other hand, were directed against strain-specific epitopes within the H5 globular head region. The latter responses were not detected in subjects that received an immunization lacking the adjuvant. Therefore, including adjuvants with seasonal influenza vaccines could potentially overcome the hurdle of high pre-existing antibodies. Indeed, data from a recent report suggest that antibody responses directed against the H1 HA stem region were readily boosted with AS03-adjuvanted 2009 pandemic H1N1 vaccine, even in subjects with high pre-existing HAI titers against the virus [47].

### 6.3. Unconventional Immunogen Platforms

Traditionally, vaccine antigens are derived from inactivated or attenuated whole bacteria and viruses [5,36]. For influenza viruses, the increased reactogenicity associated with inactivated whole virus-based vaccines led to the development of split and subunit vaccine formulations. Vaccine antigens in both of these formulations can be bound by pre-existing antibodies, potentially leading to decreased overall antibody responses. However, if the vaccine antigens are introduced in a form that is not recognizable to pre-existing antibodies, improved immune responses may result even in the presence of high concentrations of pre-existing antigen-specific antibodies in serum. One example for such a platform is the nucleoside-modified, purified mRNA encapsulated in lipid nanoparticles (mRNA-LNPs) [48,49] which demonstrated high seroconversion rates in an H10N8 influenza vaccination trial [50].

## 7. Discussion

Here, I attempted to explain how pre-existing antibodies could interfere with influenza vaccination-induced B cell responses at the epitope level. I believe that the concept of “epitope blocking” [16,17] is becoming more accepted as a mechanism of selective inhibition of responses targeting certain epitopes by pre-existing antibodies directed against these epitopes. Another angle would be blocking of responses to a particular epitope by antibodies directed against an adjacent epitope. In the latter scenario, I have proposed that the overall impact on ability of influenza vaccination-induced responses to protect will depend on the “protective capacity” profile of the blocking and blocked epitopes. The following questions remain to be addressed: (1) are all interfering antibodies pre-existing ones, or could those that are secreted by early plasmablast response also play a role? (2) Does blocking initial B cell recognition of its specific epitope early in the response have the same impact as blocking a GC B cell binding to the same epitope later? (3) Are there any true negative consequences from repeat immunization of highly immune individuals, as suggested by some recent reports [51]? (4) What is the impact of pre-existing CD4+ memory T cells on the magnitude and specificity of B cell responses to influenza vaccination? (5) What are the factors that control the durability of immune responses to vaccination? Addressing these questions will inform the rational design of influenza virus—and other—vaccines aiming at enhancing the breadth and durability of protective antibody responses.

The fact that prior influenza experience shapes the response to subsequent exposures is not something new; in his 1960 article, “On the Doctrine of Original Antigenic Sin” [1], Thomas Francis wrote:

“The antibody forming mechanisms have been highly conditioned by the first stimulus, so that later infections with strains of the same type successively enhance the original antibody to maintain it at the highest level at all times in that age group. The imprint established by the original virus infection governs the antibody response thereafter. This we have called the doctrine of original antigenic sin.”
